# The apple DNA-binding one zinc-finger protein MdDof54 promotes drought resistance

**DOI:** 10.1038/s41438-020-00419-5

**Published:** 2020-12-01

**Authors:** Pengxiang Chen, Mingjia Yan, Lei Li, Jieqiang He, Shuangxi Zhou, Zhongxing Li, Chundong Niu, Chana Bao, Fang Zhi, Fengwang Ma, Qingmei Guan

**Affiliations:** 1 State Key Laboratory of Crop Stress Biology for Arid Areas/Shaanxi Key Laboratory of Apple, College of Horticulture, Northwest A&F University, 712100 Yangling, Shaanxi P. R. China; 2 The New Zealand Institute for Plant and Food Research Limited, Hawke’s Bay, New Zealand

**Keywords:** Plant sciences, Plant stress responses

## Abstract

DNA-binding one zinc-finger (Dof) proteins constitute a family of transcription factors with a highly conserved Dof domain that contains a C2C2 zinc-finger motif. Although several studies have demonstrated that Dof proteins are involved in multiple plant processes, including development and stress resistance, the functions of these proteins in drought stress resistance are largely unknown. Here, we report the identification of the *MdDof54* gene from apple and document its positive roles in apple drought resistance. After long-term drought stress, compared with nontransgenic plants, *MdDof54* RNAi plants had significantly shorter heights and weaker root systems; the transgenic plants also had lower shoot and root hydraulic conductivity, as well as lower photosynthesis rates. By contrast, compared with nontransgenic plants, *MdDof54*-overexpressing plants had higher photosynthesis rates and shoot hydraulic conductivity under long-term drought stress. Moreover, compared with nontransgenic plants, *MdDof54*-overexpressing plants had higher survival percentages under short-term drought stress, whereas *MdDof54* RNAi plants had lower survival percentages. *MdDof54* RNAi plants showed significant downregulation of 99 genes and significant upregulation of 992 genes in response to drought, and 366 of these genes were responsive to drought. We used DAP-seq and ChIP-seq analyses to demonstrate that MdDof54 recognizes *cis*-elements that contain an AAAG motif. Taken together, our results provide new information on the functions of MdDof54 in plant drought stress resistance as well as resources for apple breeding aimed at the improvement of drought resistance.

## Introduction

Drought stress is one of the most important limiting factors for global agricultural development because it can impair crop growth and production^1^. Understanding how plants respond and adapt to drought stress is important for breeding stress-resistant crops and thus for food security worldwide. Plants have evolved a series of responses to address the adverse effects of drought stress at the morphological, physiological, and molecular levels^2,3^. When challenged with drought stress, plants exhibit several physiological responses, including drought avoidance and drought tolerance^4,5^. Under drought stress, the CO_2_ assimilation rate and photosynthesis rate are reduced, in part owing to the decreased content and activity of Calvin cycle enzymes, thereby decreasing biomass accumulation^6^. Plants absorb water and nutrients from the surrounding soil through their roots. Moreover, plants often limit their shoot growth as a response to drought stress while simultaneously enhancing their root development to absorb water in deep layers^7,8^. Hence, root system size, properties, and distribution play critical roles in plant drought resistance^9^.

Plant morphological and physiological responses to drought stress have been thoroughly studied^3^; however, the molecular mechanisms underlying drought resistance are more elusive and complex. Transcriptional regulation plays a central role in the control of plant development and responses to abiotic stress, and transcription factors (TFs) are the key factors in transcriptional regulation^10,11^. One group of TFs whose members are involved in the plant stress response are TFs with zinc-finger-binding domains that bind to DNA to activate or suppress the transcription of downstream target genes^12^. Recent research suggests that zinc-finger TFs play important roles in plant development and stress tolerance^13–19^. Zinc-finger proteins are classified into several subgroups based on the number and location of cysteine (C) and/or histidine (H) residues: C2H2 (TFIIIA), C8 (steroid-thyroid receptor), C6 (GAL4), C3HC4 (RING finger), C2HC (retroviral nucleocapsid), C2HC5 (LIM domain), C2C2 (GATA-1), C3H (Nup 475), and C4HC3 (Requiem) subgroups^20^.

Members of the Dof (DNA-binding one zinc finger) TF family have a highly conserved DNA-binding domain, the Dof domain, which contains a C2C2 zinc-finger motif^21,22^. The first Dof protein was discovered in maize (ZmDof1), and Dof proteins have subsequently been identified in other plant species, such as Arabidopsis and rice^23^, as well as in many other crop species^24–26^, including apple^27^. Dof proteins are involved in multiple plant physiological processes, including phytochrome signaling^28^, carbohydrate metabolism^29^, seed germination^30^, flowering time regulation^31^, hormone responses^32^, seed dormancy^33^, lipid synthesis^34^, floral vasculature^35^, leaf senescence^36^, resistance to powdery mildew^37^, and resistance to salt stress^38^. Although Dof family genes are widely reported to affect the physiological processes of plants, whether they play roles in plant drought resistance is largely unknown. Drought stress can stimulate the expression of several Dof genes^27^. For example, two Dof genes (*TaDof14* and *TaDof15*) were significantly induced by drought treatment in *Triticum aestivum*^39^. Nevertheless, the biological functions of Dof proteins, including Dof54, in response to drought have not been elucidated to date.

Unlike other zinc-finger proteins that contain several zinc fingers, Dof family proteins contain only one N-terminal zinc finger that is 52 amino acids in length^21^. The sequences and functions of Dof proteins from various plant species vary; however, their Dof domain sequences show high similarity, indicating that they have similar DNA-binding specificity^40^. To date, with the exception of the pumpkin Dof protein AOBP, which binds an AGTA motif, all reported Dof proteins can bind to the AAAG motif *cis*-regulatory element or its oppositely oriented sequence CCCT^16,21,40^. In addition, four maize Dof proteins have been found to bind an (A/T)AAAG sequence^41^.

Domesticated apple is the most widely grown species in the genus *Malus*. In temperate regions where apple trees are often cultivated, frequent drought stress threatens apple quality and production. A total of 60 apple Dof genes are present in the apple genome, and the majority are responsive to polyethylene glycol (PEG), NaCl, cold, and exogenous abscisic acid (ABA) treatment, suggesting that Dof proteins may participate in apple tolerance to abiotic stress^27^. Although there is an abundance of evidence to suggest that Dof family proteins play important roles in plant development and in response to powdery mildew and salt stress, whether apple Dof genes play a role in drought resistance remains unclear. In our previous research, we found that *MdDof54* is significantly induced by PEG in *Malus sieversii*, which is extremely tolerant to drought stress^42^. Here, we report that a Dof family gene from apple (*Malus* × *domestica*), *MdDof54*, plays a positive role in apple drought tolerance and adaptation.

## Results

### Identification of the apple drought-responsive gene *MdDof54*

Previously, we performed an RNA-seq analysis using drought-treated *M. sieversii*, a drought-tolerant *Malus* species. Among the differentially expressed genes (DEGs), we identified a zinc-finger TF that was dramatically induced by drought^42^. To understand the nature of this zinc-finger protein, we cloned its corresponding gene from the *M*. × *domestica* genome. Sequence analysis showed that it contained a Dof domain at its N terminus (Supplementary Fig. 1), and protein alignment showed that it had 97% sequence similarity to the sequence of the apple protein MDP0000308863, which was named MdDof54 in previous research^27^ (Supplementary Fig. 1). Because of the high heterozygosity of the apple genome, we considered that both genes were the same, and we named our protein MdDof54.

**Fig. 1:**
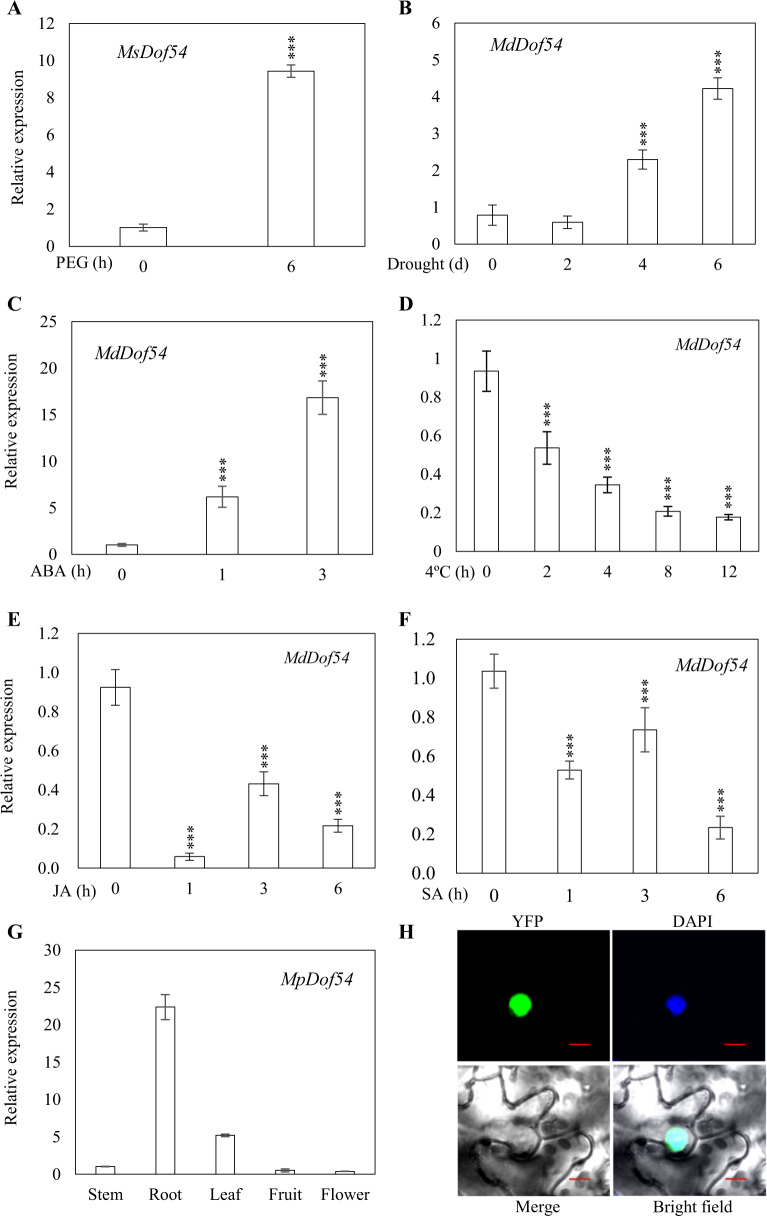
Expression patterns of *MdDof54*. **a**–**f** Expression of *MdDof54* in response to polyethylene glycol (PEG), drought stress, abscisic acid (ABA), cold stress, jasmonic acid (JA), and salicylic acid (SA). Two-month-old apple (*Malus sieversii*) plants were cultivated hydroponically for one additional month and treated with 20% PEG 8000 for 6 h (**a**). Two-month-old apple (*Malus* × *domestica*) plants grown in a greenhouse were treated with drought (**b**), low temperature (**d**), 0.1 mM ABA (**c**), 0.1 mM JA (**e**), or 0.1 mM SA (**f**) for the designated times. The asterisks indicate significant differences based on one-way ANOVA and Tukey′s test (***p < 0.001). **g** Tissue-specific expression of *MdDof54* in *Malus prunifolia*. (**h**) Subcellular localization of MdDof54. MdDof54 was fused to yellow fluorescent protein (YFP), which was then transiently expressed in the epidermal cells of tobacco leaves. The fluorescence signals of YFP and 4′,6-diamidino-2-phenylindole (DAPI) were detected by dual-channel confocal microscopy. DAPI was used to detect nuclei. Bars = 5 μm

To verify the RNA-seq results, we used quantitative real-time PCR (qRT-PCR) to examine the expression of *MsDof54* in *M. sieversii* in response to 6 h of PEG treatment. We found that *MsDof54* was significantly upregulated by PEG (Fig. 1a). To further verify the expression of *MdDof54* in domesticated apple (*M*. × *domestica*), we applied a short-term drought treatment and found that the expression level of *MdDof54* was also increased by drought (Fig. 1b). In addition, *MdDof54* was induced by ABA treatment (Fig. 1c) but repressed by cold, jasmonic acid (JA), and salicylic acid (SA) treatments (Fig. 1d–f). Tissue-specific expression analysis demonstrated that *MdDof54* was expressed predominantly in apple roots, followed by the leaves (Fig. 1g). Transient expression in tobacco leaves revealed that the MdDof54 protein was localized in the nucleus (Fig. 1h).

### Long-term drought tolerance of *MdDof54* RNAi apple plants

**Fig. 2:**
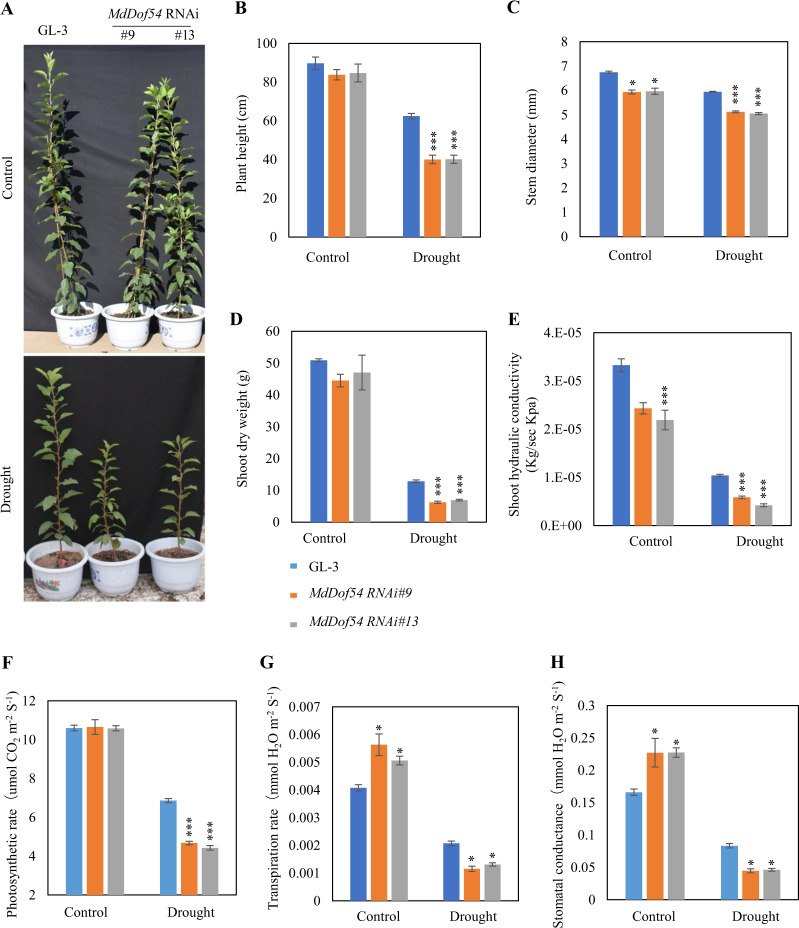
Morphology, shoot hydraulic conductivity, and photosynthesis capacity of *MdDof54* RNAi plants under long-term drought stress. Two-month-old GL-3 (transgenic apple background) and *MdDof54* RNAi plants were transferred to a greenhouse for an additional month and were then exposed to a moderate drought treatment for two months. **a**–**e** Morphological characteristics of GL-3 and *MdDof54* RNAi plants after drought stress: Images of GL-3 and *MdDof54* RNAi plants (**a**), plant height (**b**), stem diameter (**c**), shoot dry weight (**d**), and shoot hydraulic conductivity (**e**). **f**–**h** Photosynthesis capacity of GL-3 and *MdDof54* RNAi plants after drought stress: photosynthesis rate (**f**), transpiration (**h**), and stomatal conductance (**g**). The asterisks indicate significant differences between the GL-3 and transgenic lines based on one-way ANOVA and Tukey’s test (*p < 0.05; ***p < 0.001). The error bars indicate standard errors (*n* = 15)

Given that *MdDof54* was induced by drought stress, we next wondered whether MdDof54 played a role in apple drought resistance. We first generated transgenic apple plants using an RNAi approach with GL-3 (selected from progeny of Royal Gala) as the genetic background. Gene expression analysis revealed that *MdDof54* was 40–60% silenced in the *MdDof54* RNAi plants (Supplementary Fig. 2). We then transferred the *MdDof54* RNAi plants to a greenhouse and exposed them to drought stress treatment for 2 months. After the long-term drought treatment, *MdDof54* RNAi plants were significantly shorter than the GL-3 plants (Fig. 2a, b). The stem diameter of the *MdDof54* RNAi plants was also dramatically smaller than that of GL-3 plants after long-term drought (Fig. 2c). The shoot dry weights of the *MdDof54* RNAi plants were consistently lower than those of the GL-3 plants after drought (Fig. 2d), and the drought-treated *MdDof54* RNAi plants also exhibited lower shoot hydraulic conductivity (Fig. 2e). By contrast, the plant height and shoot dry weight of the *MdDof54* RNAi plants were comparable to those of GL-3 plants under control conditions (Fig. 2b, d), although their stem diameter and shoot hydraulic conductivity were clearly reduced (Fig. 2c, e). After long-term drought, the *MdDof54* RNAi plants presented reduced photosynthesis and transpiration rates and reduced stomatal conductance (Fig. 2f–h). Under the control conditions, they did not exhibit reduced rates of these parameters compared with those of the GL-3 plants (Fig. 2f–h). In addition, the leaf area of the *MdDof54* RNAi plants was smaller than that of the GL-3 plants after drought, but there was no difference in leaf area between the two genotypes under control conditions (Supplementary Fig. 3).

**Fig. 3:**
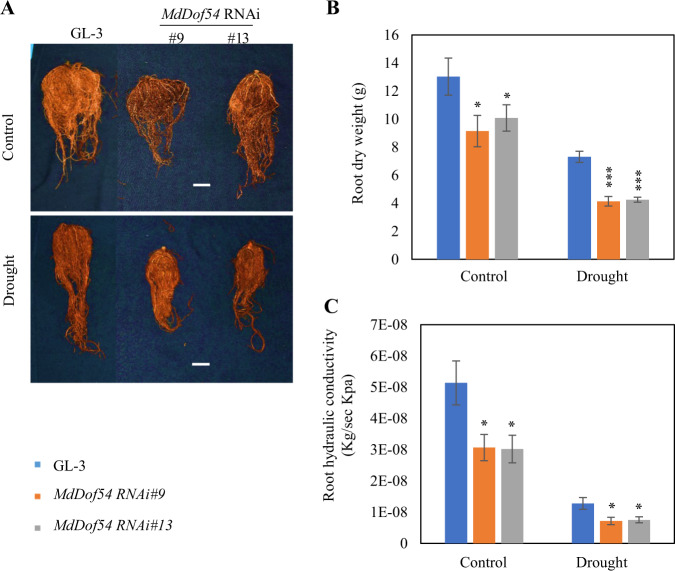
Root development and hydraulic conductivity of *MdDof54* RNAi plants under long-term drought stress. Two-month-old GL-3 and *MdDof54* RNAi plants were transplanted to a greenhouse for an additional month and then exposed to a moderate drought treatment for 2 months. **a** Morphological characteristics of roots of GL-3 and *MdDof54* RNAi plants after drought stress. Bar = 5 cm. **b**, **c** Root dry weight (**b**) and root hydraulic conductivity (**c**) of GL-3 and *MdDof54* RNAi plants after drought stress. The asterisks indicate significant differences between the GL-3 and transgenic lines based on one-way ANOVA and Tukey’s test (*p < 0.05; ***p < 0.001). The error bars indicate standard deviations (*n* = 15)

Because *MdDof54* was expressed predominantly in the roots (Fig. 1g), we next examined the root development and physiology of the *MdDof54* RNAi plants after long-term drought. Compared with the GL-3 plants, the *MdDof54* RNAi plants presented lower root dry weights and lower root hydraulic conductivity under both normal and drought conditions (Fig. 3a–c).

Taken together, the results above suggest that the *MdDof54* RNAi plants were more sensitive to long-term drought stress than were the control plants.

### Long-term drought tolerance of *MdDof54* overexpression (OE) plants

**Fig. 4:**
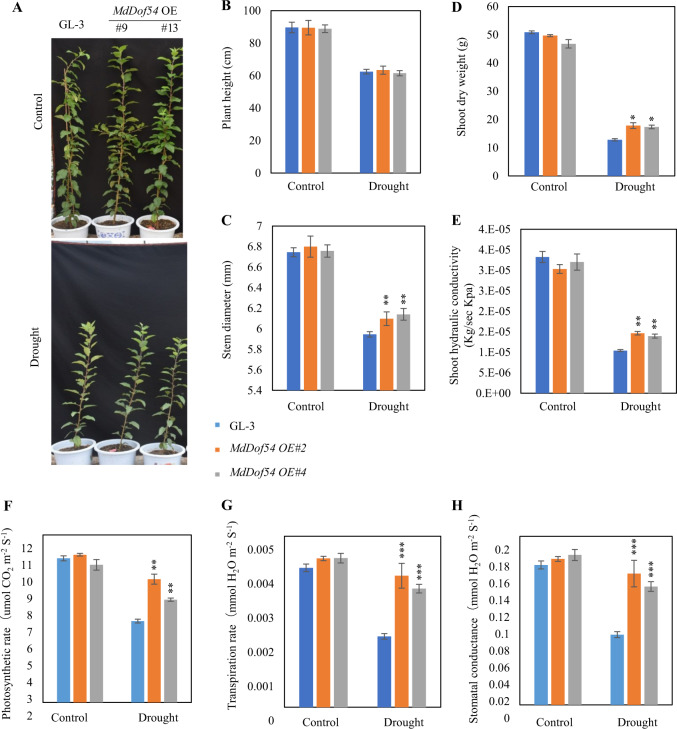
Morphology, shoot hydraulic conductivity, and photosynthesis capacity of *MdDof54* overexpression (OE) plants under long-term drought stress. Two-month-old GL-3 and *MdDof54* OE plants were transferred to a greenhouse for an additional month and were then exposed to a moderate drought treatment for two months. **a**–**e** Morphological characteristics of GL-3 and *MdDof54* OE plants after drought stress: images of GL-3 and *MdDof54* OE plants (**a**), plant height (**b**), stem diameter (**c**), shoot dry weight (**d**), and shoot hydraulic conductivity (**e**). **f**–**h** Photosynthesis capacity of GL-3 and *MdDof54* RNAi plants after drought stress: photosynthesis rate (**f**), transpiration (**h**), and stomatal conductance (**g**). The asterisks indicate significant differences between the GL-3 and transgenic lines based on one-way ANOVA and Tukey’s test (*P < 0.05; **P < 0.01; ***P < 0.001). The error bars indicate standard errors (*n* = 15)

To further confirm the regulation of drought resistance by *MdDof54*, we generated *35S:MdDof54* transgenic plants that exhibited a 10–12-fold increase in *MdDof54* expression (Supplementary Fig. 5). Under control conditions, the *MdDof54*-OE plants did not differ from the GL-3 plants in terms of height, stem diameter, shoot dry weight, or shoot hydraulic conductivity (Fig. 4a–e). After long-term drought stress, although the genotypes did not differ in height, compared with the GL-3 plants, the *MdDof54* OE plants had greater stem diameter, shoot dry weight, and shoot hydraulic conductivity (Fig. 4a–e). Under long-term drought, the *MdDof54* OE plants also had a higher photosynthesis rate, transpiration rate, and stomatal conductance (Fig. 4f–h); in addition, after long-term drought, the *MdDof54* OE plants had a larger leaf area (Supplementary Fig. 6). We also investigated the root development of the *MdDof54* OE plants after long-term drought. There were no differences in root dry weight or root hydraulic conductivity between the *MdDof54* and GL-3 plants (Supplementary Fig. 7). Taken together, these data suggest that *MdDof54* OE plants are tolerant to long-term drought.

### Short-term drought resistance of *MdDof54* transgenic plants

**Fig. 5:**
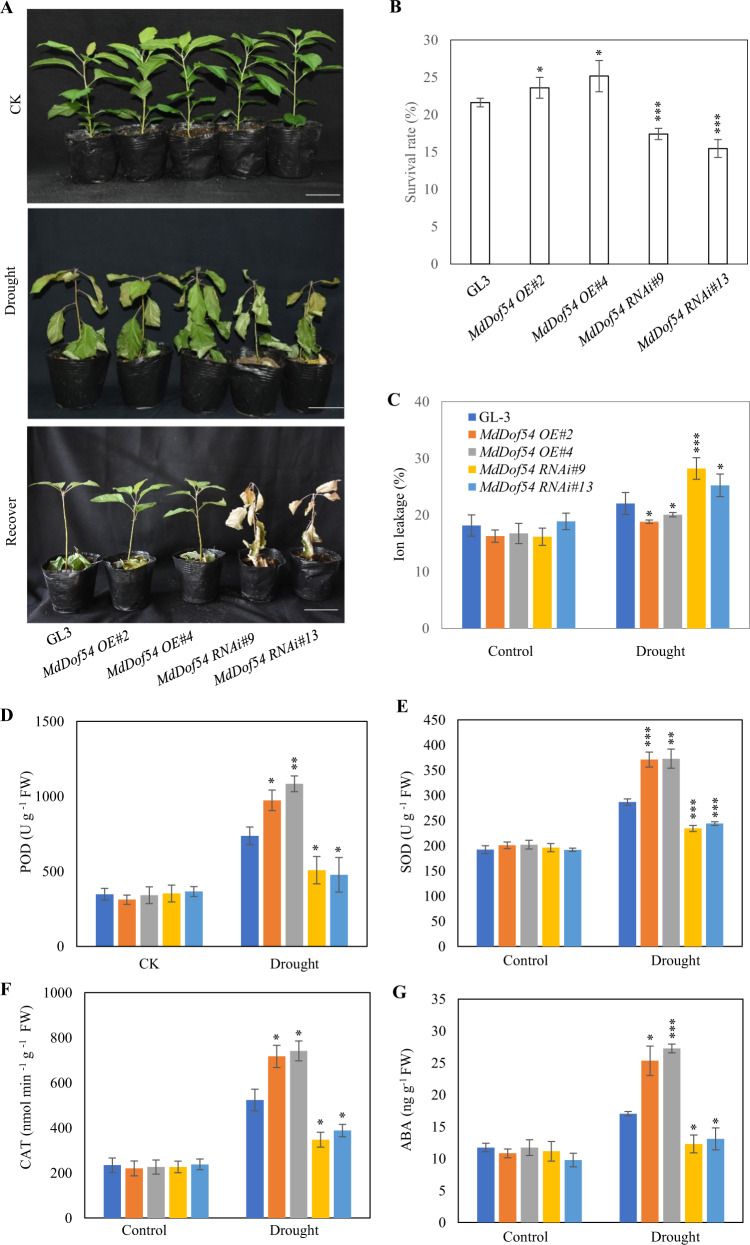
Drought tolerance of *MdDof54* transgenic plants under short-term drought. **a** Morphological characteristics of GL-3, *MdDof54* OE, and *MdDof54* RNAi plants in response to short-term drought stress. Two-month-old plants were transplanted into soil for two additional months and then exposed to drought stress. After 34 days of drought stress, the plants were allowed to recover at saturated soil moisture for 7 days, after which the recovery results were obtained. Bars = 5 cm. **b** Survival percentage of plants shown in **a** after the recovery period. **c**–**g** Physiological changes in GL-3, *MdDof54* OE, and *MdDof54* RNAi plants after drought stress: ion leakage (**c**), peroxidase (POD) activities (**d**), superoxide dismutase (SOD) activities (**e**), catalase (CAT) activities (**f**), and ABA content (**g**). The asterisks indicate significant differences between the GL-3 and transgenic lines based on one-way ANOVA and Tukey′s test (*p < 0.05; **p < 0.01; ***p < 0.001). The error bars indicate standard deviations [*n* = 20 in **b**, 5 in **c**–**g**]. OE overexpression

To verify the biological function of *MdDof54* in apple under drought stress, we performed a short-term drought stress experiment using the *MdDof54* RNAi and OE plants. After a drought treatment of 34 days followed by 7 days of recovery, compared with the GL-3 plants, the *MdDof54* OE plants presented a greater survival percentage, and *MdDof54* RNAi plants presented a lower survival percentage (Fig. 5a, b). Moreover, compared with the GL-3 plants, the *MdDof54* OE plants showed lower electrolyte leakage under short-term drought stress, and the *MdDof54* RNAi plants showed higher electrolyte leakage (Fig. 5c). We also measured peroxidase (POD), superoxide dismutase (SOD), and catalase (CAT) activities and determined the ABA content in the GL-3 and *MdDof54* transgenic plants in response to drought (Fig. 5d–g). The results showed that, compared with the GL-3 plants, the *MdDof54* OE plants had higher POD, SOD, and CAT activities, as well as a higher ABA content, under drought. However, the ABA content and POD, SOD, and CAT activities were lower in the *MdDof54* RNAi plants than in the GL-3 plants in response to drought (Fig. 5d–g). These results further support the conclusion that MdDof54 is a positive regulator of apple drought resistance.

### Gene expression profiling of *MdDof54* RNAi plants under drought stress

*MdDof54* is a Dof zinc-finger TF and therefore likely mediates the expression of multiple target genes. To further understand the molecular function of *MdDof54* in the response to drought stress, we performed an RNA-seq analysis with *MdDof54* RNAi plants and GL-3 plants. Whole-genome expression analysis revealed 1619 drought-responsive genes in the GL-3 plants (Supplementary Data 1). Under control conditions, 358 genes were upregulated and 335 genes were downregulated in the *MdDof54* RNAi plants compared with the GL-3 plants (Supplementary Data 2). Similarly, under drought conditions, 992 genes were upregulated and 99 genes were downregulated in the *MdDof54* RNAi plants compared with the GL-3 plants (Supplementary Data 3). Among the 1091 DEGs in the *MdDof54* RNAi plants under drought stress, 366 were drought-responsive genes (Supplementary Data 4). Gene Ontology (GO) analysis of the drought-stressed *MdDof54* RNAi plants revealed that the DEGs were involved in water deprivation as well as JA, SA, and ethylene responses. The drought treatment also significantly enriched the expression of genes associated with cell death and with the response to chitin and organonitrogen compounds in the *MdDof54* RNAi plants (Supplementary Fig. 4).

**Fig. 6:**
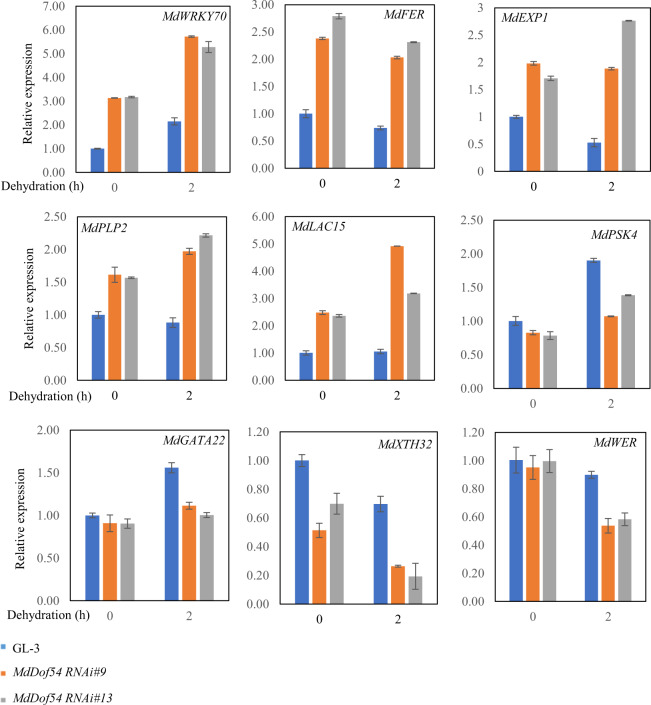
Verification of differentially expressed genes in *MdDof54* RNAi plants under drought stress. Abscised apple leaves from two-month-old GL-3 and *MdDof54* RNAi plants were dehydrated for 0 or 2 h. The error bars indicate standard deviations (*n* = 3)

We selected ten genes for qRT-PCR verification of the RNA-seq results. Similar expression levels were determined with both techniques for nine of the ten genes: *WRKY DNA-BINDING PROTEIN 70* (*MdWRKY70*), *FERONIA* (*MdFER*), *EXPANSIN 1* (*MdEXP1*), *PATATIN-LIKE PROTEIN* 2 (*MdPLP2*), *LACCASE 5* (*MdLAC5*), *GATA TRANSCRIPTION FACTOR 22* (*MdGATA22*), *XYLOGLUCAN ENDOTRANSGLUCOSYLASE/HYDROLASE 32* (*MdXTH32*), *WEREWOLF 1* (*MdWER*), and *PHYTOSULFOKINE 4 PRECURSOR* (*MdPSK4*) (Fig. 6). Among these genes, homologs of *MdPSK4* are positive regulators of the drought response^43^, while *MdWRKY70*, *MdFER*, and *MdPLP2* are negative regulators of the drought response^44–47^. The results shown in Fig. 6 demonstrate that *MdDof54* negatively regulates the expression of *MdWRKY70*, *MdPLP2*, *MdLAC5*, *MdEXP1*, and *MdFER* and positively regulates the expression of *MdGATA2*2, *MdXTH32*, *MdWER*, and *MdPSK4*.

### Potential targets of MdDof54 in apple

**Fig. 7:**
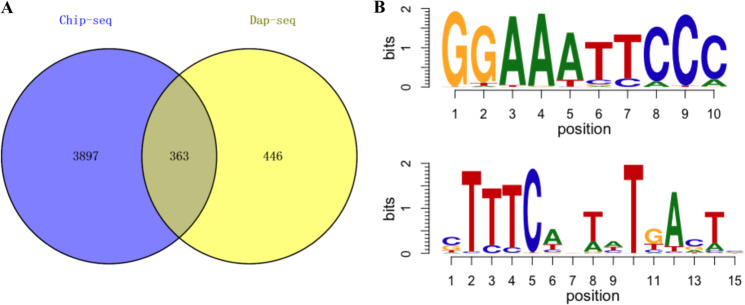
Target genes and recognition sites of MdDof54. **a** Venn diagrams of chromatin immunoprecipitation sequencing (ChIP-seq) and DNA affinity purification sequencing (DAP-seq) analysis of MdDof54. The numbers indicate target genes using ChIP-seq or DAP-seq analysis. A native antibody against MdDof54 was used to pull down DNAs bound by MdDof54. **b** The two potential recognition motifs of MdDof54 revealed by DAP-seq. Recombinant MdDof54 proteins were used to perform DAP-seq

Dof family TFs usually bind to AAAG elements in the promoters of their targets. To identify potential targets of MdDof54 in the apple genome, we first performed a chromatin immunoprecipitation sequencing (ChIP-seq) analysis using an anti-MdDof54 antibody and identified 4260 potential targets (Supplementary Data 5). To further identify potential targets, we carried out a DNA affinity purification sequencing (DAP-seq) analysis, which revealed 809 potential targets of MdDof54 (Supplementary Data 6). When the results of the two analyses were combined, 363 of the potential targets overlapped (Fig. 7a) (Supplementary Data 7), suggesting that they were more likely to be direct targets of MdDof54. Using DAP-seq analysis, we identified two motifs with the highest score, GGAAA and TTTC, which is consistent with the findings of previous reports (Fig. 7b)^16,21,40,41^. Among the 363 likely targets were genes that encoded key enzymes involved in plant growth and the abiotic stress response, such as *ACC OXIDASE 1* (*MdACO1*), *ASCORBATE PEROXIDASE 3* (*MdAPX3*), *TRIGALACTOSYL DIACYLGLYCEROL 2* (*MdTGD2*), and the gene that encodes the large subunit of ADP-glucose pyrophosphorylase (*MdAPL3*), which catalyzes the initial rate-limiting step in starch biosynthesis.

## Discussion

In this research, we identified a zinc-finger protein, MdDof54, that was significantly induced by drought stress in apple. We found that *MdDof54* plays a positive role in apple drought tolerance by mediating drought-responsive gene expression and modulating shoot and root development and photosynthesis.

We previously found that *MdDof54* was significantly induced by simulated drought treatment^42^, implying that MdDof54 may play a role in apple drought tolerance. In the current research, several lines of evidence support the assertion that MdDof54 is a positive regulator of apple drought tolerance. First, under long-term drought stress, compared with the GL-3 plants, the *MdDof54* RNAi plants were shorter and had reduced shoot and root hydraulic conductivity (Figs. 2 and 3). In addition, the *MdDof54* RNAi plants had lower photosynthesis capacity under long-term drought stress (Fig. 2). Second, compared with the GL-3 plants, the *MdDof54* OE plants had higher photosynthesis capacity and shoot hydraulic conductivity under drought conditions (Fig. 4). Third, using a short-term drought treatment, we found that, compared with the GL-3 plants, the *MdDof54* RNAi plants had a lower survival percentage, whereas the *MdDof54* OE plants had a higher survival percentage (Fig. 5). Fourth, under drought stress, the *MdDof54* OE plants had higher POD, SOD, and CAT activities, while the *MdDof54* RNAi plants presented lower levels of POD, SOD, and CAT activities. Antioxidant enzymes such as CAT, POD, and SOD play important roles in removing excessive accumulation of reactive oxygen species (ROS), which cause damage to the plant cell membrane system^48,49^. Fifth, compared with the GL-3 plants, the *MdDof54* OE plants contained more ABA under drought stress, whereas the *MdDof54* RNAi plants had less ABA (Fig. 5). Taken together, these data suggest that MdDof54 is a positive regulator of drought tolerance.

We noticed that *MdDof54* OE plants were affected by drought at varying degrees for the different traits. For example, after long-term drought stress, compared with the GL-3 plants, the *MdDof54* OE plants had greater stem diameter, shoot dry weight, and shoot hydraulic conductivity, but not plant height (Fig. 4a–e). Furthermore, the *MdDof54* OE plants had a higher photosynthesis rate, transpiration rate, stomatal conductance, and leaf area (Fig. 4f–h and Supplementary Fig. 6). We also measured POD, SOD, and CAT activities and ABA content under short-term drought treatment. Compared with the GL-3 plants, the *MdDof54* OE plants showed higher ABA content and antioxidant enzyme activities (Fig. 5d–g). Therefore, we conclude that the *MdDof54* OE plants were tolerant to apple drought stress, although their root dry weight and root hydraulic conductivity did not show any differences with those of the GL-3 plants after drought stress (Supplementary Fig. 7).

Root development and root hydraulic conductivity play important roles in plant drought tolerance^50–52^. In our study, compared with the GL-3 plants, the *MdDof54* RNAi plants had lower root dry weight and lower root hydraulic conductivity after long-term drought stress (Fig. 3), indicating a positive role for MdDof54 in the regulation of root development and root water transport. However, the *MdDof54* OE plants did not have higher root dry weight or higher root hydraulic conductivity after drought stress (Supplementary Fig. 6). This may have occurred because increased *MdDof54* expression in the OE plants was not high enough to stimulate root development and root water transport.

Biomass production has been correlated with leaf area duration under drought^53^. Under drought treatment, compared with the GL-3 plants, the *MdDof54* RNAi plants had lower leaf area (Supplementary Fig. 3). This reduction in leaf area, together with decreased stomatal conductance and transpiration, probably contributed to the reduced photosynthesis rate of the *MdDof54* RNAi plants under drought (Fig. 2 and Supplementary Fig. 3). As a result, the decreased photosynthesis rates adversely affected growth parameters such as plant height, stem diameter, and root growth (Figs. 2 and 3). On the other hand, the increased leaf area, stomatal conductance, and transpiration of the *MdDof54* OE plants led to an elevated photosynthesis rate under drought (Fig. 4 and Supplementary Fig. 6). However, a higher photosynthesis rate may have resulted in the allocation of photosynthates primarily to stem growth in *MdDof54* OE plants, resulting in greater stem diameter but no changes in plant height or the root system (Fig. 4 and Supplementary Fig. 7). It is possible that, although *MdDof54* was overexpressed in these plants, the increase in assimilated carbon products was not sufficient to promote increased allocation to the root system.

The photosynthesis rate in higher plants depends on both stomatal opening and metabolite concentrations, including levels of ribulose-1,5-biphosphate carboxylase/oxygenase (rubisco)^6,54^. Because of decreased stomatal conductance, drought stress often lowers CO_2_ assimilation and photosynthesis capacity^6^. We found that the photosynthesis rate, transpiration rate, and stomatal conductance of the *MdDof54* RNAi plants were lower than those of the GL-3 plants after 2 months of drought stress, whereas the same parameters were higher in the *MdDof54* OE plants under the same treatment (Figs. 2 and 4). However, under well-watered conditions, we also noticed that the transpiration rates and stomatal conductance of the *MdDof54* RNAi plants were higher than those of the GL-3 plants, although both genotypes had comparable rates of photosynthesis (Fig. 2). This implied that, under conditions of sufficient water, *MdDof54* RNAi plants need more water to assimilate the same amount of CO_2_ as GL-3 plants assimilate. In addition, we found that the shoot and root hydraulic conductivities of the *MdDof54* RNAi plants were lower than those of the GL-3 plants under well-watered conditions (Figs. 2 and 3), indicating that the limited hydraulic conductivity might restrict the overall growth and photosynthesis of the *MdDof54* RNAi plants.

From the RNA-seq data, we identified 1091 DEGs in the *MdDof54* RNAi plants under drought stress. GO analysis indicated that these DEGs were involved in the regulation of the ROS metabolic process and SA and JA responses, as well as cell death and the response to chitin and organonitrogen compounds (Supplementary Fig. 4). Among the 1091 DEGs, 99 were downregulated in the *MdDof54* RNAi plants under drought stress, while 992 were upregulated (Supplementary Data 4), suggesting that the main role of MdDof54 under drought may be to repress gene expression. Homologs of *WRKY70*, *MdFER*, and *MdPLP2* have been shown to be negative regulators of drought tolerance^4–47^, and PSK4 promotes plant growth and responds to biotic and abiotic stress^43^. We found that *MdPSK4* was downregulated in the *MdDof54* RNAi plants under drought treatment (Fig. 6). The decreased growth and drought tolerance of the *MdDof54* RNAi plants under drought may reflect decreased expression of *MdPSK4* and increased expression of other genes, including *MdWRKY70*, *MdFER*, and *MdPLP2* (Fig. 6).

The reported Dof proteins often recognize the AAAG motif in their target promoters^16,21,40,41^. In this study, we used two approaches to identify the direct targets of MdDof54. We first performed ChIP-seq analysis using an anti-MdDof54 antibody and identified 4260 potential targets (Supplementary Data 5). We then used a DAP-seq assay with recombinant MdDof54 proteins and identified 809 potential targets (Supplementary Data 6). After combining the results from both approaches, we identified 363 targets (Fig. 7). Among these targets was a homolog of TGD2, a transmembrane lipid transfer protein localized on the photosynthetic membranes. TGD2 is involved in the synthesis of thylakoid glycolipids and can hinder photosynthesis efficiency^55,56^. An APL3 homolog that encodes a large subunit of ADP-Glc pyrophosphorylase (AGPase) was also identified. APL3 is a key enzyme in starch synthesis^57,58^. From RNA-seq data, we found that *MdAPL3* was downregulated in the *MdDof54* RNAi plants under drought stress (Supplementary Data 3). Decreased *MdAPL3* levels may contribute to insufficient amounts of carbon products in the *MdDof54* RNAi plants, leading to their reduced height under drought conditions.

In summary, we characterized the positive roles of MdDof54 in apple drought resistance. Under long-term drought stress, MdDof54 facilitated root development, stomatal conductance, transpiration, photosynthesis, and hydraulic conductivity, which lead to improved growth of apple trees. The expression of a number of genes, such as *MdWRKY70*, was regulated by MdDof54 and may also contribute to the increased drought resistance of apple trees.

## Methods

### Vector construction and generation of transgenic plants

To generate constructs for subcellular localization, the CDS of *MdDof54* was cloned into the pEarleyGate 104 vector and fused to YFP, resulting in YFP-MdDof54, which was then transformed into *Agrobacterium* C58C1. C58C1 carrying YFP-MdDof54 was transiently expressed in tobacco leaves according to the method described by Xie et al.^59^. Fluorescent signals in transformed tobacco leaves were detected with an FV1200 confocal microscope (Olympus, Japan).

To obtain transgenic apple plants, the coding region of *MdDof54* was cloned into a pGWB418 OE vector. To generate RNAi lines, 200 bp of the *MdDof54* coding region was inserted into the RNAi-mediated vector pK7WIWG2D. *Agrobacterium tumefaciens*-mediated transformation was subsequently performed as described previously^60^. GL-3, the seedlings of which were selected from a Royal Gala (*M*. × *domestica*) population and exhibit high regeneration capability, was used as the genetic background^60^.

The primers used for constructing all the vectors are shown in Supplementary Table 1.

### Drought stress treatment

To investigate the expression pattern of *MdDof54*, two-month-old apple (*M*. *sieversii*) plants were cultivated hydroponically for an additional month and treated with 20% PEG 8000 for 6 h^42^. In addition, 2-month-old apple (*M*. × *domestica*) plants were transferred to a greenhouse and cultivated for an additional month before being subjected to drought (irrigation was withheld for up to 6 days) or low temperature (4 °C) for designated times or being sprayed with ABA (0.1 mM), SA (0.1 mM), and JA (0.1 mM)^61^.

To study tissue-specific expression, newly formed roots, stems from newly produced shoots, mature leaves, flowers, and young fruits (30 days after blooming) were collected from five-year-old apple (*M*. *prunifolia*) trees that planted in an orchard at the Horticulture Experimental Station of Northwest A&F University, Yangling, Shaanxi, China (34°16′N, 108°4′E)^61^.

For long-term drought treatment, rooted GL-3, transgenic *MdDof54* RNAi, and OE plants were grown in pots (30 cm × 18 cm) filled with potting media (Pindstrup, Denmark) and locally obtained loess sandy soil. After 2 months, the plants were then placed in a greenhouse under natural conditions, with a temperature of 20–35 °C and a humidity of 35–55%. After another month, plants of each genotype were divided into two groups: a well-watered group (*n* = 15) and a long-term drought treatment group (*n* = 15). The plants of the well-watered group were watered daily such that a field capacity of 75–85% was maintained, whereas the plants from the long-term drought treatment group were maintained at field capacity of 45–55% by the weighing method described by Guo et al.^62^. This process lasted for 2 months. After drought treatment, plant height was measured by the use of a meter stick, and stem diameter was measured by a Vernier caliper. The photosynthesis rate, transpiration rate, and stomatal conductance were measured with an LI-6400XT portable photosynthesis system (LI-COR, USA) on sunny days from 7 a.m. to 9 a.m. (the temperature was 26 ± 2 °C). The parameters for the measurement were set as follows: the photosynthesis photon flux density was 1000 μmol m^−2^ s^−1^, the airflow rate was a constant 500 μmol s^−1^, and the concentration of CO_2_ in the cuvette was 400 cm^3^ m^−3^. Data were collected from the mature leaves (the fifth to eighth leaf from the base of the plant stems). The leaf area of the same leaves was measured by using automatic computational software in conjunction with a scanner (Perfection V19, Epson, Japan). Root and shoot hydraulic conductivities were determined with a third-generation high-pressure flow meter (Dynamax, Inc., USA) as described previously^52^. Roots and shoots were collected and washed for the dry weight measurements after the tissue had completely dried at 65 °C.

For short-term drought treatment, the plants were grown in a growth chamber under long-day conditions (16 h:8 h, light:dark) at 21 °C and an irradiance of 4000 lx for 2 months, after which they were subjected to short-term drought (*n* = 20). Before treatment, the plants were fully irrigated, and each pot was brought to the same weight (denoted as day 0). Watering was withheld for 34 days, and the survival percentage was determined after seven days of rehydration. On the 23rd day of the short-term drought treatment, plants with the same growth vigor from the control group and the drought treatment group were selected, leaf discs (diameter = 8 mm) were used to measure conductivity, and the data were recorded as R1. The leaf discs were then boiled for 30 min, and conductivity was measured and recorded as R2. Electrolyte leakage was calculated as the percentage of R1 out of R2.

### Measurement of ABA and activities of POD, SOD, and CAT

Reagent kits from Conmin Biotechnology Company (Suzhou, China) were used to measure POD (#POD-1-Y), SOD (#SOD-1-W), and CAT (#CAT-1-W) activities according to the manufacturer’s protocols. ABA was extracted as previously described^63^ with extraction buffer (methanol:isopropanol:acetic acid = 20:79:1, v:v:v). The ABA content was measured by a QTRAP^®^ 5500 LC-MS/MS (AB SCIEX, USA).

### RNA extraction and qRT-PCR

Total leaf RNA was isolated according to a previously described method^64^. qRT-PCR-based analyses were carried out based on previous research methods^65^. The primers used are shown in Supplementary Table 1. All the experiments were repeated, with three biological replicates.

### RNA-seq data analysis

Abscised apple leaves, which were air dried for 0 or 2 h, from 2-month-old GL-3 and *MdDof54* RNAi plants were used for RNA extraction. The RNA was subjected to sequencing on the Illumina HiSeq platform by Novogene (Beijing, China), and the sequences were mapped to the *M*. × *domestica* genome from the NCBI database by HISAT2 (ref. ^66^). Differences in gene expression were analyzed by DESeq2 (ref. ^67^), with a threshold of *p* values below 0.05 and log2(fold change) greater than 1.5. The fragments per kilobase of transcript per million fragments mapped (FPKM) values were calculated by Cufflinks^68^. GO annotation and enrichment were analyzed by the online tools agriGO^69^ and KOBAS^70^.

### DAP-seq data analysis

An Amp-DAP library was constructed as described previously^71^. In brief, genomic DNA was extracted from GL-3 by the CTAB method, and the RNA was eliminated by RNase A (EN0531, Thermo Fisher, USA). Afterward, 5 μg of DNA was sonicated to approximately 200 bp by Bioruptor Plus (Diagenode, USA) and was then subjected to end-repair using a Fast DNA End Repair Kit (K0771, Thermo Scientific), according to the manufacturer’s instructions. The end-repaired DNA was polyadenylated using Klenow fragment (M0212S, NEB, UK) and dATP (N0440S, NEB), followed by ligation of the annealed Y adaptor (adaptor strand A, 5′-ACACTCTTTCCCTACACGACGCTCTTCCGATCT-3′; adaptor strand B, 5′-P-GATCGGAAGAGCACACGTCTGAACTCCAGTCAC-3′, where “P” indicates a 5′ phosphate group) by T4 DNA ligase (M0202S, NEB). The library was amplified by Phusion High-Fidelity DNA Polymerase (M0530S, NEB) in conjunction with primer A (AATGATACGGCGACCACCGAGATCTACACNNNNNNNNACACTCTTTCCCTACACGACGCTCTTCCGATCT) and primer B (CAAGCAGAAGACGGCATACGAGATNNNNNNNNGTGACTGGAGTTCAGACGTGTGCTCTTCCGATC), where “NNNNNNNN” represents the 8mer index sequence for multiplexing, yielding an amp-DAP library.

To obtain recombinant and purified MdDof54 proteins in vitro, the full-length coding sequence of MdDof54 was inserted into a pGEX4-T vector. The MdDof54 protein was expressed in *Escherichia coli* and purified by Pierce™ Glutathione Magnetic Agarose Beads (Thermo Fisher). The empty vector was also purified to obtain GST proteins.

One hundred microliters of MagneGST Glutathione Particles (V8611, Promega, USA) was washed with MagneGST Binding/Wash Buffer and resuspended in the buffer to a final volume of 300 μL in a 1.5 mL EP tube. The purified protein was then added to bind the MagneGST glutathione particles at 4 °C for 30 min. The protein-bead complex was washed four times at room temperature (each time for 5 min), followed by resuspension in 300 μL of buffer. Approximately 100 ng of the amp-DAP library was added to the resuspended solution, with gentle agitation at 4 °C for 1 h, followed by washing four times to remove free DNA. Finally, the DNA was resuspended in 100 μL of elution buffer, boiled at 98 °C for 10 min, amplified, and separated via agarose gel electrophoresis. DNA fragments 200–400 bp in length were subsequently recovered from the agarose gel via a GeneJET Extraction and DNA Cleanup Micro Kit (K0832, Thermo Fisher).

The recovered DNA was subjected to paired-end sequencing (150 bp) on an Illumina TruSeq platform (Novogene). The sequences were trimmed by Trimmomatic to filter low-quality reads and adaptors. The clean reads were then aligned to the latest *M*. × *domestica* genome from the NCBI database using the short-read mapping software Bowtie2 (ref. ^72^). Each output file underwent bam conversion, followed by sorting and indexing with SAMtools^73^. A control sample was added for background subtraction during peak calling using MACS2 (ref. ^74^). Enriched motifs were ultimately produced with findMotifGenome.pl of HOMER^75^.

### ChIP-seq analysis

The specific anti-MdDof54 antibody was synthesized by Genscript (Nanjing, China). The ChIP-seq methods and data analysis were the same as those of previous research^59^. Briefly, tissue-cultivated apple leaves grown on Murashige and Skoog (MS) culture media for 4 weeks were cross‐linked in 1% formaldehyde. After chromatin isolation and sonication, ChIP-grade protein A/G magnetic beads (26162, Thermo Fisher) were used to preclear the chromatin supernatant. Anti-MdDof54 antibodies were then added, after which the sample was incubated overnight at 4 °C. A no-antibody sample was used as a control. The immune complexes were then collected with protein A/G magnetic beads and washed with high-/low-salt solutions, an LiCl solution, and TE buffer before being eluted with elution buffer. Reverse crosslinking was carried out by incubation at 65 °C in 5 M NaCl for 8 h. The proteins were digested by 10 mg mL^−1^ proteinase K for 1 h at 45 °C, followed by sequencing of the recovered DNA on the Illumina TruSeq platform (Novogene).

The sequences were analyzed using the same process as that used for DAP-seq.

### Statistical analysis

The experimental data were analyzed with SPSS 20.0 software. One-way ANOVA was used to compare significant differences based on Tukey’s test (*p* < 0.05, *p* < 0.01, or *p* < 0.001), and the error bars indicate standard deviations, unless otherwise noted.

## Supplementary information

Supplementary Table 1

Supplemental figures

Data set 1

Data set 2

Data set 3

Data set 4

Data set 5

Data set 6

Data set 7

## Data Availability

All the sequencing data in this study have been submitted to the NCBI database under accessions PRJNA613874 (RNA-seq), PRJNA613876 (ChIP-seq), and PRJNA613877 (DAP-seq).
