# Supplementary material for: The apple DNA-binding one zinc-finger protein MdDof54 promotes drought resistance
Source: Hortic Res. 2020 Dec 1;7:195. doi: 10.1038/s41438-020-00419-5 (PMC7704620; doi:10.1038/s41438-020-00419-5)
Supplement: Supplementary file 1 — Supplementary Table 1 [file 41438_2020_419_MOESM1_ESM.pdf]

Supplemental Table 1. Primers used in this study.

Primer Name	Primer Sequence (5' to 3')	Purpose
MdDof54 qPCR F	CCAGGCTCTCACAGACTGAC	qRT-PCR analysis
MdDof54 qPCR R	CAGGGGTAGTGGAGTTGTGG	
MdDof54 full lenght F	ATGTTCACAGATCCTACTGTTAAACAAAT	Clone full-length gene
MdDof54 full lenght R	CATAGATCGTGAGAAAACTTCAAACC	
MdDof54 RNAi F	GGGGACAAGTTTGTACAAAAAAGCAGGCTGCACCACAA CTCCACTACCCCT	Plasmid construction for MdDof54 RNAi plants
MdDof54 RNAi R	GGGGACCACTTTGTACAAGAAAGCTGGGTCTTGCACCAC TTGGTCTTCGT	
MdDof54 OE F	GGGGACAAGTTTGTACAAAAAAGCAGGCTGCATGTTCAC AGATCCTACTGTTAAACAAAT	Plasmid construction for MdDof54 OE plants
MdDof54 OE R	GGGGACCACTTTGTACAAGAAAGCTGGGTCCATAGATCG TGAGAAAACTTCAAACC	
MdDof54 attb ns F	GGGGACAAGTTTGTACAAAAAAGCAGGCTGCATGTTCAC AGATCCTACTGTTAAACAAAT	Plasmid construction for MdDof54 subcellular localization
MdDof54 attb ns R	GGGGACCACTTTGTACAAGAAAGCTGGGTCAGATCGTGA GAAAACTTCAAACC	
MdWRKY70-qpcr-F	GCCCTGATCTTTGGCCATCT	qRT-PCR analysis
MdWRKY70-qpcr-R	AATGGACGACGAGGATGAGC	
MdFER-qpcr-F	GGGCGTATTAAGGTCGATGGGGATG	qRT-PCR analysis
MdFER-qpcr-R	GATGAGGTTTGGGTGGCGGAGC	
MdEXP1-qpcr-F	ACTCTTTCCTCAACGGCCAG	qRT-PCR analysis
MdEXP1-qpcr-R	AGTTTATGGTGTTGTTCAGAACTGT	
MdPLP2-qpcr-F	TTGAAGAAGCCGGTGTCACG	qRT-PCR analysis
MdPLP2-qpcr-R	GCCTAATTAAGCTTCTTTCCTTCCG	
MdLAC15-qpcr-F	CCACCCAGAATGCCACCTTG	qRT-PCR analysis
MdLAC15-qpcr-R	AGGCCTAGATCATGCCCACA	
MdPSK4-qpcr-F	ACATGTCGTCCAAGCTCACC	qRT-PCR analysis
MdPSK4-qpcr-R	CGTCATGCCGGATAATGCAAG	
MdGATA22-qpcr-F	GCTGAAGCTGCAATCCTCCT	qRT-PCR analysis
MdGATA22-qpcr-R	GCAAAACCACAGAAAAGGAACGA	
MdXTH32-qpcr-F	TGCTTCACAGATTGGGAGTTC	qRT-PCR analysis
MdXTH32-qpcr-R	TTGGCAAGTAATGACCCATCTC	
MdWER-qpcr-F	TAAAGTCACAGAGGTGCGGC	qRT-PCR analysis
MdWER-qpcr-R	TCAAGGACCATCTGTTGCCA	
MdDof54-EcoRIp42a-F	GGATATCGGGGATCCGAATTCATGTTCACAG ATCCTACTGTTAAACAAAT	Plasmid construction for MdDof54 Expressing protein in vitro
MdDof54-SalIp42a-R	GAGTGCGGCCGCAAGCTTGTCGACAGA TCGTGAGAAAACTTCAAACC	
