# Supplementary material for: The apple DNA-binding one zinc-finger protein MdDof54 promotes drought resistance
Source: Hortic Res. 2020 Dec 1;7:195. doi: 10.1038/s41438-020-00419-5 (PMC7704620; doi:10.1038/s41438-020-00419-5)
Supplement: Supplementary file 2 — Supplemental figures [file 41438_2020_419_MOESM2_ESM.pdf]

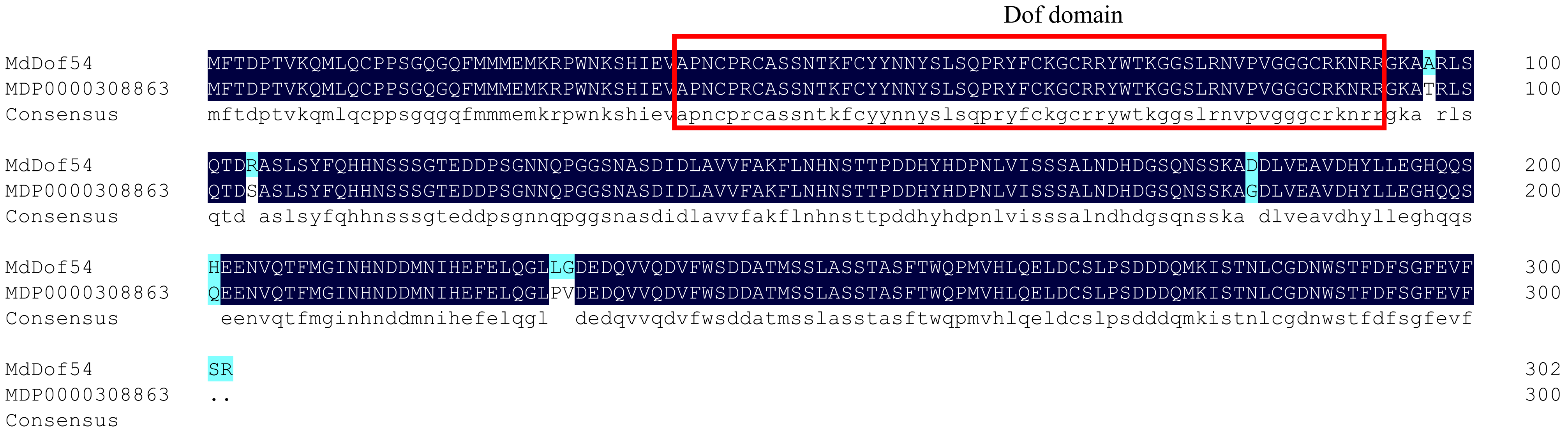

Supplementary Figure 1. Protein alignment of MdDof54 from apple genome. The Dof domain is indicated with a red box.

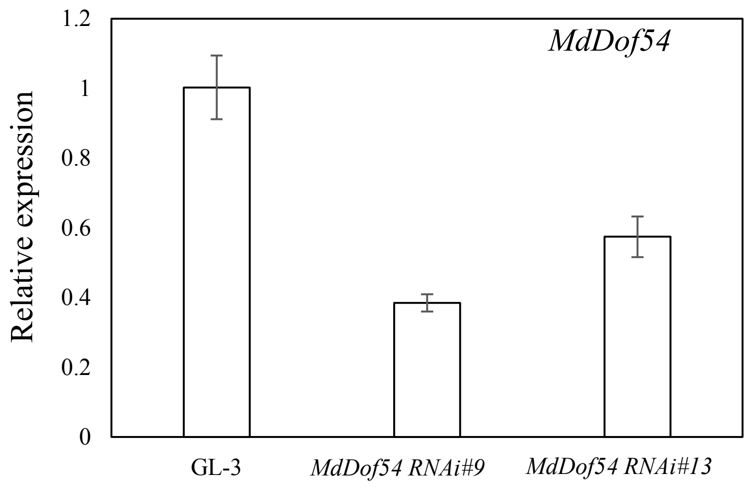

Supplementary Figure 2. Generation of *MdDof54* RNAi plants. *MdDof54* mRNA levels were detected in the *MdDof54* RNAi plants. Error bars indicate standard deviation (n = 3).

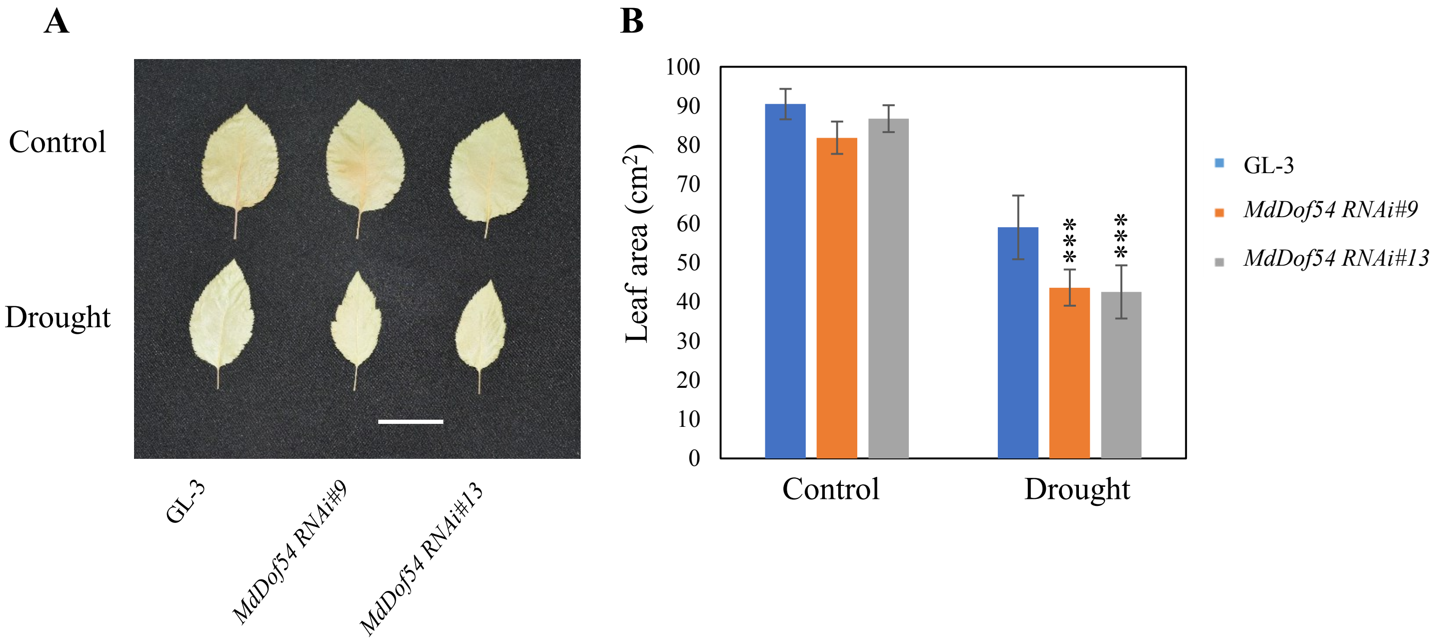

Supplementary Figure 3. Leaf area of *MdDof54* RNAi plants under long-term drought stress. Two-month-old plants were transferred to a greenhouse for one additional month and then exposed to a moderate drought treatment for two months. Oven-dried leaf (A) and leaf area (B) of GL-3 and *MdDof54* RNAi plants after drought stress. Asterisks indicate significant differences between GL-3 and transgenic lines based on one-way ANOVA and Tukey's test (*, $p < 0.05$; **, $p < 0.01$; ***, $p < 0.001$). Error bars indicate standard deviation ($n = 15$).

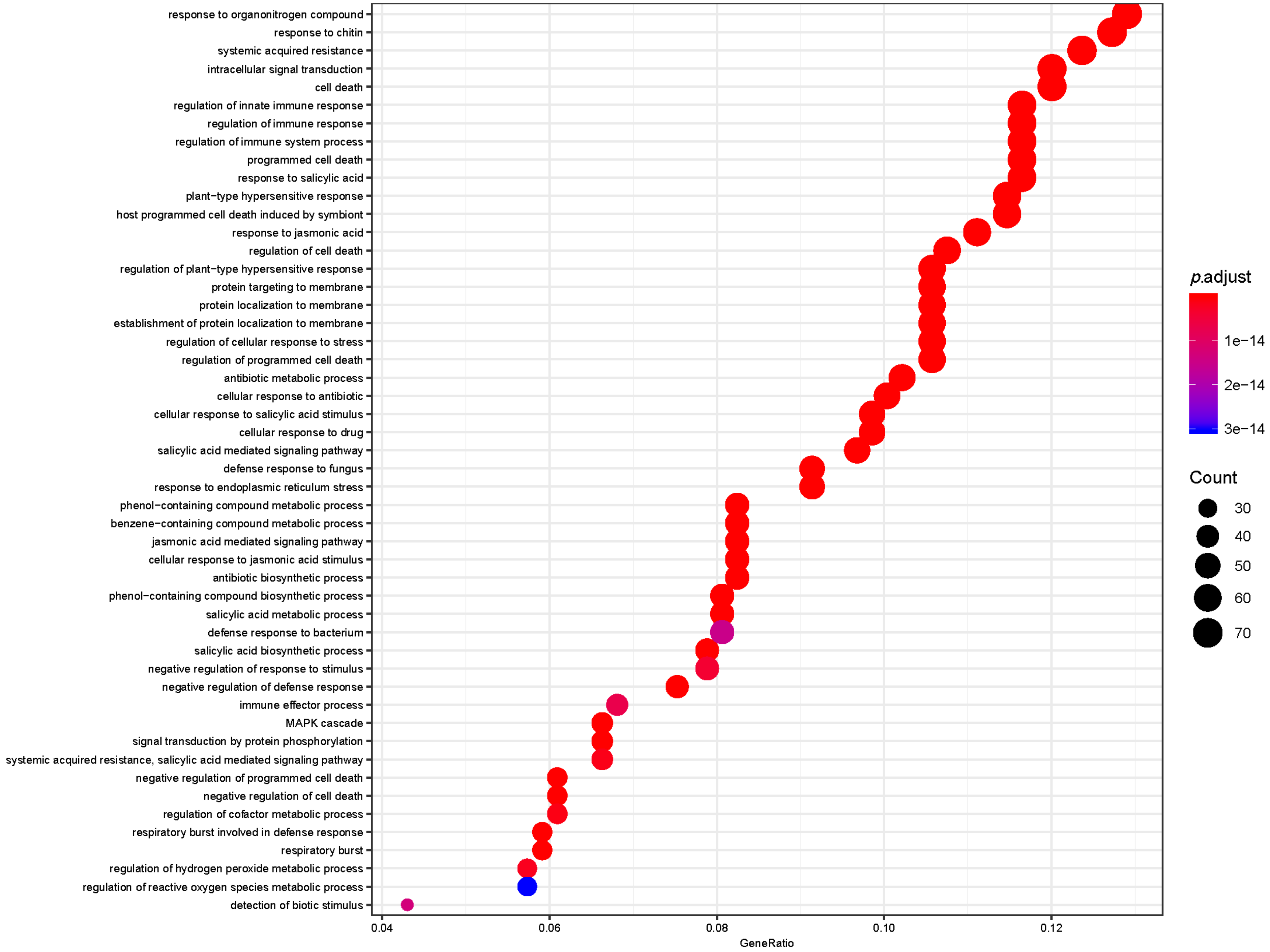

Supplementary Figure 4. Gene Ontology (GO) analysis of differentially expressed genes in *MdDof54* RNAi plants under drought stress. The adjusted *p*-values indicate which pathways are more likely to have biological significance. The red dots indicate low adjusted *p*-values (high enrichment), and the blue dots indicate high adjusted *p*-values (low enrichment). Gene Ratio indicates the ratio of gene numbers annotated to a pathway among Supplementary data 3. Count: count of genes.

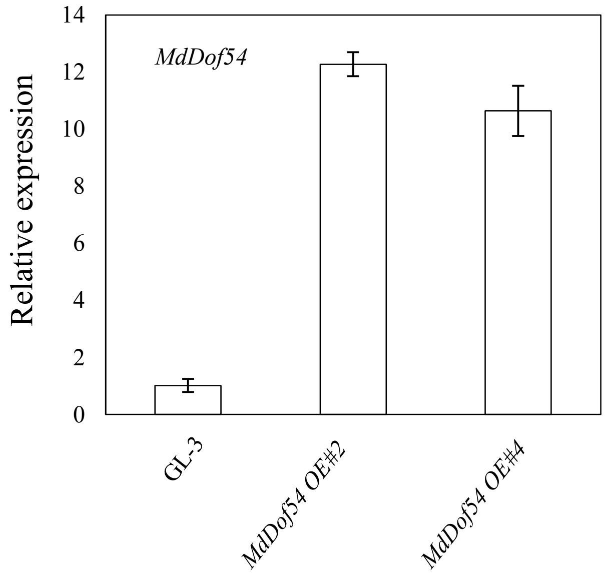

Supplementary Figure 5. Generation of *MdDof54* overexpression plants. *MdDof54* mRNA levels were detected in the *MdDof54* overexpression plants. Error bars indicate standard deviation (*n* = 3).

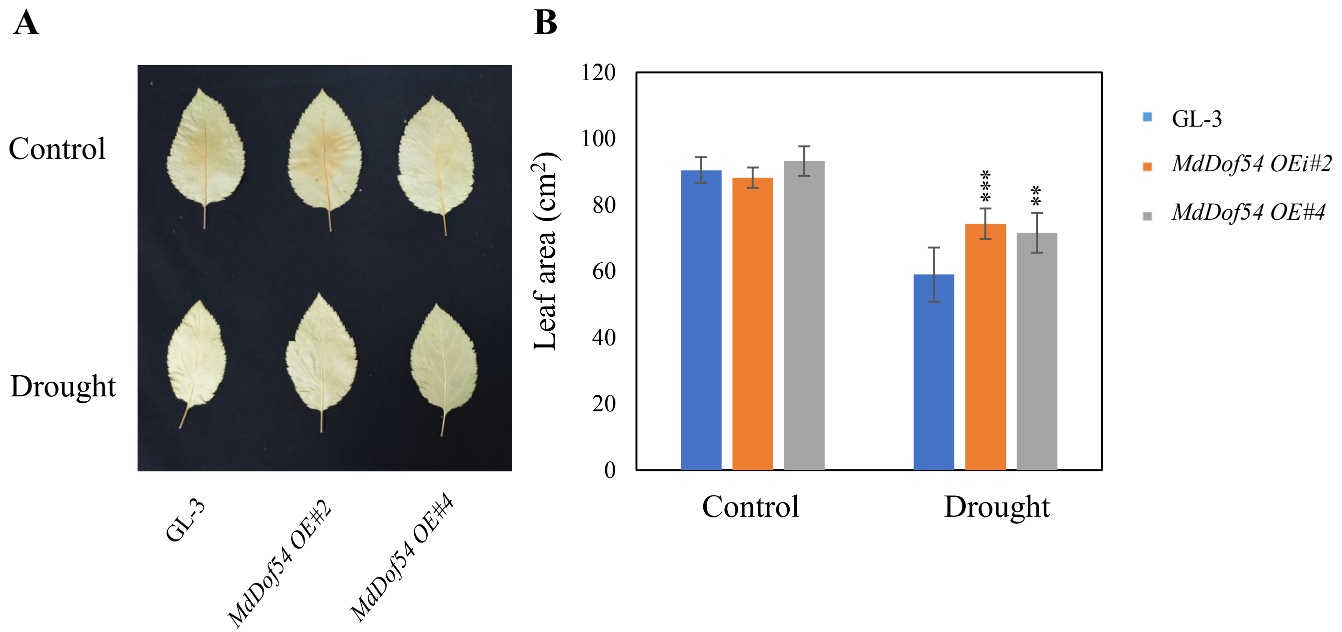

Supplementary Figure 6. Leaf area of *MdDof54* overexpression plants under drought. Two-month-old plants were transferred to a greenhouse for one additional month and then exposed to a moderate drought treatment for two months. Oven dried leaf (A) and leaf area (B) of GL-3 and *MdDof54* OE plants. Error bars indicate standard errors (n = 15). Asterisks indicate significant differences between GL-3 and transgenic lines based on one-way ANOVA and Tukey's test (*, $p < 0.05$; **, $p < 0.01$; ***, $p < 0.001$). Error bars indicate standard deviation. OE, overexpression.

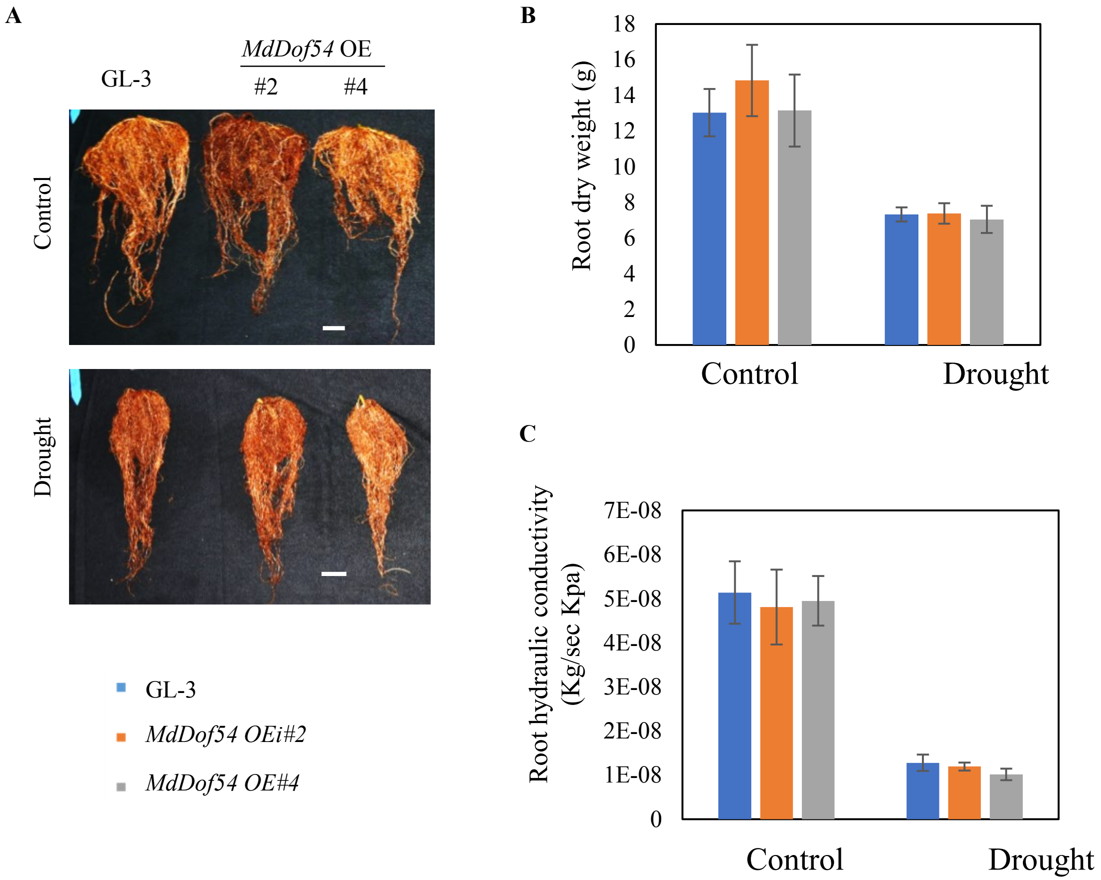

Supplementary Figure 7. Root development and hydraulic conductivity of *MdDof54* OE plants under long-term drought stress. Two-month-old plants were transferred to a greenhouse for one additional month and then exposed to a moderate drought treatment for two months. (A) Morphological characteristics of roots from GL-3 and *MdDof54* RNAi plants after drought stress. Bar = 5cm. (B)–(C) Root dry weight (B) and root hydraulic conductivity (C) of GL-3 and *MdDof54* OE plants after drought stress. Asterisks indicate significant differences between GL-3 and transgenic lines based on one-way ANOVA and Tukey's test (*, $p < 0.05$; **, $p < 0.01$; ***, $p < 0.001$). Error bars indicate standard deviation ($n = 15$). OE, overexpression.
